# Inhibition of pancreatic cancer cell growth in vitro by the tyrphostin group of tyrosine kinase inhibitors.

**DOI:** 10.1038/bjc.1993.491

**Published:** 1993-12

**Authors:** J. Gillespie, J. F. Dye, M. Schachter, P. J. Guillou

**Affiliations:** Academic Surgical Unit, St. Mary's Hospital Medical School, Imperial College of Science, Technology and Medicine, London, UK.

## Abstract

**Images:**


Br. J. Cancer (1993), 68, 1122–1126 © Macmillan Press Ltd., 1993

### Inhibition of pancreatic cancer cell growth *in vitro* by the tyrphostin group of tyrosine kinase inhibitors

J. Gillespie[1], J.F. Dye[1], M. Schachter[2] & P.J. Guillou[1]

[1]*Academic Surgical Unit and* [2]*Department of Clinical Pharmacology, St. Mary's Hospital Medical School, Imperial College of Science, Technology and Medicine, London W2 1NY, UK.*

**Summary** Tyrphostins are a group of low molecular weight synthetic inhibitors of protein tyrosine kinases (PTK). The intracellular domains of the receptors for epidermal growth factor (EGF), transforming growth factor-α (TGF-α), insulin-like growth factor 1 (IGF-1) possess PTK activity. Since EGF, TGF-α and IGF-1 are considered to play an important role in the proliferation of pancreatic cancer cells, we studied the effects of tyrphostins on the growth of three human pancreatic cancer cell lines (MiaPaCa-2, Panc-1 and CAV). The tyrphostins AG17, T23 and T47 all inhibited EGF and serum-stimulated DNA synthesis. AG17 was found to be the most potent of these agents and caused a dose-dependent but reversible inhibition of cell growth. Furthermore using an immunoblotting procedure we also found AG17 to inhibit EGF-induced tyrosine phosphorylation in the MiaPaCa-2 cell line. Tyrosine kinase inhibitors may prove to be useful agents for the treatment of pancreatic cancer.

It has been proposed that in ductal pancreatic cancer cells the production of certain growth factors, particularly epidermal growth factor (EGF), transforming growth factor-α (TGF-α) and insulin-like growth factor 1 (IGF-1), together with the overexpression of their receptors, leads to unregulated cellular proliferation (Korc *et al.*, 1986; Smith *et al.*, 1987; Chen *et al.*, 1990; Ohmura *et al.*, 1990; Barton *et al.*, 1991; Lemoine *et al.*, 1992). These growth factors bind to specific membrane receptors whose intracellular domains possess intrinsic protein tyrosine kinase (PTK) activity which is activated after ligand binding and is essential for signal transduction and biological activity. Interference with this pathway may provide a means of designing effective therapy for pancreatic cancer.

The tyrphostins are a recently described family of low molecular weight protein tyrosine kinase inhibitors (Gazit *et al.*, 1989; Levitzki, 1990). The characteristic active pharmacophore of these compounds is the hydroxy-cis-benzylidene-malono-nitrile moiety (Gazit *et al.*, 1991). These compounds have been demonstrated to be competitive inhibitors of EGF, insulin and platelet derived growth factor (PDGF) receptor kinases (Lyall *et al.*, 1989; Bryckaert *et al.*, 1992) and the protein kinases $p60^{src}$, $p210^{bcr\text{-}abl}$, $p185^{bcr\text{-}abl}$, $p140^{abl}$ (Anafi *et al.*, 1992). In addition, tyrphostins have been shown to be effective blockers of EGF and PDGF-dependent proliferation in various cells (Yaish *et al.*, 1988; Bilder *et al.*, 1991). Some of these agents exhibit selectivity and can discriminate between, for example, the EGF and insulin receptors or the PDGF and EGF receptors (Yaish *et al.*, 1988; Bryckaert *et al.*, 1992). More remarkably some tyrphostins can discriminate between the EGF receptor and the closely related product of the *erb*B-2/*neu* oncogene, structures that are 80% homologous in the kinase domain (Gazit *et al.*, 1991). It is considered that this selectivity occurs because the tyrphostins compete for the substrate site of the kinase, rather than the ATP binding site, and therefore, since different PTKs possess different substrate sites it is possible to design selective PTK inhibitors for each PTK (Levitzki, 1990; Levitzki & Gilon, 1991). The tyrphostins have been tested against a number of serine/threonine kinases and have been found to be inactive (Gazit *et al.*, 1989; Yaish *et al.*, 1988).

Because of the importance of tyrosine kinases in the growth factor induced control of pancreatic cancer cell growth, our aim was to examine the effect of the tyrphostins T23, T47 and AG17 on the proliferation of three human ductal pancreatic cancer cell lines. On finding that AG17 was the most potent inhibitor of proliferation, we sought evidence that this effect was indeed mediated via the inhibition of PTK activity by studying phosphotyrosine levels within the MiaPaCa-2 cell line using an immunoblotting technique.

Correspondence: P.J. Guillou, Academic Surgical Unit, St. Mary's Hospital Medical School, London, W2 1NY, UK.
Received 1 June 1993; and in revised form 11 August 1993.

### Materials and methods

### *Protein tyrosine kinase inhibitors*

Tyrphostin AG17 (RG50872) was a generous gift from Professor A. Levitzki (Department of Biological Chemistry, The Hebrew University of Jerusalem, Israel). Tyrphostins T23 and T47 were purchased from Biomol Research Laboratories. Stock solutions of the agents were prepared in dimethyl sulfoxide (DMSO) and diluted to appropriate concentrations in culture medium prior to the addition to the cells. An equivalent dilution of DMSO without the inhibitor served as a control.

### *Quantification of tumour cell growth*

MiaPaCa-2 and Panc-1 are both poorly differentiated human ductal pancreatic carcinoma cell lines and were obtained from the European Cell Culture Collection. CAV was derived from a moderately differentiated ductal carcinoma and was kindly provided by Dr D. Beauchamp (Department of Surgery, University of Texas Medical Branch, Galveston, USA). MiaPaCa-2 and Panc-1 were routinely maintained as monolayers in Dulbecco's Modified Eagle's Medium (DMEM)(ICN/Flow) containing 10% foetal calf serum (FCS). CAV cells were grown in 50% RPMI/50% DMEM containing 5% FCS.

For the measurement of DNA synthesis, tumour cells were harvested by trypsinisation and after washing were plated out in 96 well plates at $5 \times 10^4$ cells ml$^{-1}$ (for serum stimulation) or $1 \times 10^5$ cells ml$^{-1}$ (for EGF stimulation) in serum free medium (DMEM only) for 24 h. After this period of growth arrest the medium was supplemented with $10^{-8}$ M EGF (Gibco) or 10% FCS (Globepharm), these concentrations having been determined as optimal in preliminary experiments and reported elsewhere (Gillespie *et al.*, 1992). Varying concentrations of AG17 (ranging from $10^{-5}$ M to $10^{-7}$ M), tyrphostin T23 or tyrphostin T47 (both ranging from $10^{-4}$ M to $10^{-6}$ M) were added to these cultures. The cells were incubated for 48 h at 37°C. DNA synthesis was assessed for the final 8 h by adding 0.5 μCi $^3$H-thymidine/well. The cells were then collected onto filter mats using a semi-automatic harvester (Inotech, Switzerland). Scintillation fluid was added to individual filter discs and the cell associated radioactivity counted in a beta counter (Packard 1900CA Tricarb).

For cell growth experiments the cells were again harvested by trypsinisation, washed and plated out in 24 well plates at $3 \times 10^4$ cells $ml^{-1}$ (for serum stimulation) or $8 \times 10^4$ cells $ml^{-1}$ (for EGF stimulation) in serum-free medium (SFM) for 24 h. After this period of serum starvation the medium was supplemented with $10^{-8}$ M EGF or 10% FCS with or without tyrphostin AG17. The cells were incubated at 37°C and cell numbers were determined using a Coulter Counter, at timed intervals following the initiation of culture.

### *Immunoblotting*

MiaPaCa-2 cells were plated out in six well dishes in DMEM containing 10% FCS and allowed to grow for 3 days. Prior to the addition of AG17, the cells were incubated in SFM for 24 h. Following the preincubation with AG17 (24 h) the cells were stimulated with EGF ($10^{-8}$ M) for 15 min. These cells were then lysed with sample buffer (0.06 M Tris containing 2% SDS, 100 mM DTT, 100 μM sodium orthovanadate, 10% glycerol, 0.001% bromophenol blue, pH 6.8) and boiled for 5 min. The protein concentration of the cell lysate was determined using a bicinchoninic acid kit (Pierce, Rockford, USA). Samples were stored at −70°C until analysis.

The Pharmacia Phast system was used for the electrophoresis of cell lysate samples (10–15% gradient polyacrylamide gels) and semi-dry electroblotting onto PVDF membrane. The membrane was then processed according to the following schedule: blocking carried out in PBS/0.05% Tween-20 containing sheep serum (10%), polyethylene glycol (5%), human IgG (10%) for 1 h, followed by incubation with anti-phosphotyrosine antibody directly labelled with horseradish peroxidase, PY-20 (ICN/Flow) (1 μg $ml^{-1}$) for 1 h and finally washed in 50 mM Tris/0.15 M NaCl/0.05% Tween-20 (1 × 15 min; 4 × 5 min). The membrane was then immersed in enhanced chemiluminescent reagent (Amersham) for 1 min and tyrosine phosphorylated protein bands were detected by photographic exposure of autoradiographic film for 30 s to 30 min.

### Results

### *Inhibition of EGF and serum-stimulated pancreatic cancer cell DNA synthesis by T23, T47 and AG17*

In three separate experiments, DNA synthesis was stimulated by EGF and FCS in all three of the cell lines. Furthermore, it can be seen from Table I that the amount of $^3$H-thymidine incorporated into these cell lines under the designated control conditions (serum free medium alone (SFM), SFM plus $10^{-8}$ M EGF or SFM plus 10% FCS) at fixed cell densities was consistent with small standard errors.

The tyrphostins T23 and T47 caused a dose-dependent inhibition of DNA synthesis in the two poorly differentiated cell lines MiaPaCa-2 and Panc-1 when cultured with $10^{-8}$ M EGF or 10% FCS (Figures 1 and 2). The moderately differentiated CAV cell line was only made available to us at a later date but once again it can be seen from Figure 3 that

**Figure 1** Inhibition of EGF-stimulated (**a**) and serum-stimulated (**b**) MiaPaCa-2 ■ and Panc-1 □ DNA synthesis by T23, as measured by $^3$H-thymidine incorporation. Results are expressed as a percentage of control (EGF or serum-stimulated cells) and are the mean ± s.e.m. of three separate experiments in which five determinations were made. $^*P<0.0001$ *vs* control (Student's unpaired *t*-test).

AG17 also inhibited the DNA synthesis of all three pancreatic cancer cell lines in a dose-dependent manner.

Further scrutiny of the data represented in Figures 1, 2 and 3 demonstrates two additional features in the tyrphostin-induced inhibition of DNA synthesis in these cell lines. First, bearing in mind the consistency of the measurement of $^3$H-thymidine uptake in the control cultures, on a molar basis the tyrphostin AG17 appeared to be a consistently more potent inhibitor of proliferation than did T23 or T47 by a factor of between 10 and 100-fold. For this reason AG17 was selected for all subsequent experiments. Second, it can be seen that the amount of any of the tyrphostins required to produce an equivalent amount of inhibition of tumour cell proliferation was greater during culture in 10% FCS than it was during culture in SFM containing $10^{-8}$ M EGF.

### *Inhibition of EGF and serum-stimulated pancreatic cancer cell growth by AG17*

The effect of AG17 on EGF and serum-stimulated cell growth was assessed directly by counting cell number on days 2, 3 and 6 for EGF-stimulated cells and on days 1, 2 and 3 for serum-stimulated cells since the cells grow more

**Table I** $^3$H-thymidine uptake in pancreatic cancer cell lines

| *FCS* | *SFM*[a] | *$10^{-8}$ EGF* | *SFM*[b] | *10% FCS* |
|---|---|---|---|---|
| MiaPaCa-2 (*n* = 15) | 3060 ± 281 | 112017 ± 432 | 1247 ± 36 | 42148 ± 892 |
| Panc-1 (*n* = 15) | 1713 ± 231 | 6621 ± 566 | 1069 ± 50 | ·25471 ± 892 |
| CAV (*n* = 15) | 2418 ± 59 | 11119 ± 586 | 1343 ± 129 | 16793 ± 713 |

Data expressed as mean (± s.e.m.) counts per minute. [a]Serum-free medium-base line level in EGF experiments. [b]Serum free medium-base line level in 10% FCS experiments. Differences in counts per minute between SFM[a] and SFM[b] is due to a greater number of cells being plated out in the EGF experiment (see Materials and methods).

**Figure 2** Inhibition of EGF-stimulated (a) and serum-stimulated (b) MiaPaCa-2 ■ and Panc-1 □ DNA synthesis by T47, as measured by $^3$H-thymidine incorporation. Results are expressed as a percentage of control (EGF or serum-stimulated cells) and are the mean ± s.e.m. of three separate experiments in which five determinations were made. $^*P<0.0001$, $P<0.05$ *vs* control (Student's unpaired *t*-test).

**Figure 3** Inhibition of EGF-stimulated (a) and serum-stimulated (b) MiaPaCa-2 ■, Panc-1 □ and CAV ▤ DNA synthesis by AG17, as measured by $^3$H-thymidine incorporation. Results are expressed as a percentage of control (EGF or serum-stimulated cells) and are the mean ± s.e.m. of three separate experiments in which five determinations were made. $^*P<0.0001$, $\wedge\ P<0.05$ *vs* control (Student's unpaired *t*-test).

**Figure 4** Inhibition of EGF-stimulated MiaPaCa-2 (a), Panc-1 (b) and Cav (c) cell growth by AG17. Each point is the mean of triplicate determinations. The standard errors have been omitted for clarity, but in all cases they were less than 10% of the mean.

slowly in serum free medium containing EGF only. In all three cell lines AG17 inhibited pancreatic cancer cell growth in a time and dose-dependent manner (Figures 4 and 5). AG17 appeared not to be toxic to any of the pancreatic cancer cells studied as measured by the ability of the cells to exclude trpyan blue (all cultures contained >95% viable cells at termination of culture).

The ability of cells to recover after treatment with AG17 was also investigated. Figure 6 demonstrates that the growth-inhibitory effect of the tyrphostin on the MiaPaCa-2 cell line was reversible following replacement of AG17-containing medium with fresh medium.

### *Inhibition of EGF-induced tyrosine phosphorylation by AG17 in the MiaPaCa-2 cell line*

It has previously been shown that tyrphostins are potent inhibitors of tyrosine kinase activity in a variety of non-pancreatic cells *in vitro* (Lyall *et al.*, 1989; Bryckaert *et al.*, 1992; Reddy *et al.*, 1992). To confirm that tyrphostin AG17 can inhibit tyrosine phosphorylation in pancreatic cancer cells, we examined the effect of AG17 on EGF-stimulated MiaPaCa-2 cells, which contain relatively high numbers of EGF receptors (Korc *et al.*, 1986). After a 15 min exposure of these cells to EGF, immunoblotting analysis using an anti-phosphotyrosine antibody demonstrated a dramatic increase in tyrosine phosphorylation of multiple proteins.

Figure 5 Inhibition of serum-stimulated MiaPaCa-2 (a), Panc-1 (b) and Cav (c) cell growth by AG17. Each point is the mean of triplicate determinations. The standard errors have been omitted for clarity, but in all cases they were less than 10% of the mean.

Figure 6 Reversibility of the effects of AG17 on pancreatic cancer cells. MiaPaCa-2 cells were grown in DMEM with 10% serum and either $10^{-5}$ M, $5 \times 10^{-6}$ M or $2.5 \times 10^{-6}$ M AG17. Cells were counted on days 1, 2 and 3. On day 3, medium was changed and the cells split into groups. Three groups were grown in the continued presence of AG17 (control cells) and the cells in the other groups washed and given fresh medium containing no AG17.

Figure 7 Inhibition of EGF-induced tyrosine phosphorylation in the MiaPaCa-2 cell line. U = unstimulated cells; S = EGF-stimulated cells; 1 = $10^{-5}$ M AG17; 2 = $10^{-6}$ M AG17; 3 = $5 \times 10^{-7}$ M AG17; 4 = $10^{-7}$ M AG17.

AG17 inhibited this phosphorylation, notably of a 170 kD band which corresponds to the molecular weight of the EGF receptor (Figure 7). The dose dependent effect of AG17 on this phosphorylation appeared to be similar with that seen on proliferation.

### Discussion

Several reports have demonstrated that in ductal pancreatic cancer cells tyrosine kinase activity is elevated and is considered to be related to the autocrine production of certain growth factors and the overexpression of their receptors which possess intrinsic tyrosine kinase activity (Korc *et al.*, 1986; Smith *et al.*, 1987; Ohmura *et al.*, 1990; Barton *et al.*, 1991; Lemoine *et al.*, 1992). This knowledge provides an opportunity to target a specific component of the autocrine loop in pancreatic cancer cells with a view to therapeutic manipulation. In this paper, although all three tyrphostins inhibited pancreatic cancer cell replication, we have shown that AG17 was the most potent inhibitor of EGF and serum-stimulated DNA synthesis and growth in pancreatic cancer cells by a mechanism that appears largely dependent on the inhibition of protein tyrosine kinases. In keeping with other published data using different analogues, the effects of these agents seemed principally cytostatic and reversible when the drug was removed from the culture medium.

The effect of tyrphostin AG17 (RG50872) on platelet derived growth factor (PDGF) and EGF-induced DNA synthesis in rabbit vascular smooth muscle cells has been previously examined and AG17 was found to be more specific for PDGF than for an equieffective dose of EGF ($IC_{50}$ PDGF 0.04 μM, EGF 0.22 μM) (Bilder *et al.*, 1991). It is unlikely that our pancreatic cancer cell lines express receptors for PDGF since Beauchamp *et al.* (1990) have shown that PDGF had no effect on the growth of MiaPaCa-2 and Panc-1 *in vitro*. However in our pancreatic cancer DNA synthesis studies we have found AG17 to be 10–100 times more potent than the commercially available tyrphostins T23 and T47. It was for this reason that all further work was conducted using AG17 only.

AG17 is a much less effective inhibitor of serum-induced DNA synthesis and cell growth compared with EGF-stimulated proliferation (Figures 3–5). This weaker inhibition of serum-dependent growth was expected since not only is EGF present in low concentrations in serum but also several other, mostly unidentified, growth factors in serum possibly stimulate growth through other tyrosine kinase receptors and/or other pathways not involving tyrosine kinases.

Since tyrphostin AG17 had such a dramatic effect on EGF-stimulated pancreatic cancer cell growth we also investigated the effect of AG17 on tyrosine phosphorylation in one of the cell lines. As predicted, when cells were pretreated with AG17 and then stimulated with EGF the phosphotyrosine level of multiple proteins was reduced. One particular protein was markedly affected by AG17 and since the molecular weight is approximately 170 kD we propose this to be the EGF receptor. However we are currently re-blotting our samples with anti-EGF receptor antibody to test this. Another important observation from the immunoblotting analysis is that the concentrations of AG17 needed to inhibit tyrosine phosphorylation were similar to those required for inhibition of EGF-stimulated DNA synthesis and cell growth.

Our data show that the tyrosine kinase inhibitor AG17 can inhibit the growth of pancreatic cancer cells. Recently several tyrosine kinase inhibitors have been shown to inhibit *in vivo* growth, in nude mice, of a human squamous cell carcinoma. Moreover, the combination of tyrphostins with suboptimal doses of monoclonal antibodies to the EGF receptor have been found to potentiate one another in inhibiting the growth of such tumours (Yoneda *et al.*, 1991). Regarding toxicity, preliminary data from this group suggest that tyrphostin-treated animals actually increased their appetite and weight during the 4 weeks of tyrphostin treatment, suggesting that these agents may not be particularly toxic. Clearly, however, pharmacokinetic and detailed toxicology studies are now required. Taking this into account, the present data raise the possibility that tyrphostins may prove to be useful new agents for the treatment of pancreatic cancer and other tumours in which there is increased tyrosine kinase activity.

### References

ANAFI, M., GAZIT, A., GILON, C., BEN-NERIAH, Y. & LEVITZKI, A. (1992). Selective interactions of transforming and normal abl proteins with ATP, tyrosine-copolymer substrates and tyrphostins. *J. Biochem. Chem.*, **267**, 4518–4523.

BARTON, C.M., HALL, P.A., HUGHES, C.M., GULLICK, W.J. & LEMOINE, N.R. (1991). Transforming growth factor alpha and epidermal growth factor in human pancreatic cancer. *J. Pathol.*, **163**, 111–116.

BEAUCHAMP, R.D., LYONS, R.M., YANG, E.Y., COFFEY, R.J.& MOSES, H.L. (1990). Expression of and response to growth regulatory peptides by two human pancreatic carcinoma cell lines. *Pancreas*, **4**, 369–380.

BILDER, G.E., KRAWIEC, J.A., MCVETY, K., GAZIT, G., GILON, C., LYALL, R., ZILBERSTEIN, A., LEVITZKI, A., PERRONE, M.H. & SCHREIBER, A.B. (1991). Tyrphostins inhibit PDGF-induced DNA synthesis and associated early events in smooth muscle cells. *Am. J. Physiol.*, **260**, C721–730.

BRYCKAERT, M.C., ELDOR, A.M., FONTENAY, M., GAZIT, A., OSHEROV, N., GILON, C., LEVITZKI, A. & TOBELEM, G. (1992). Inhibition of Platelet-derived growth factor-induced mitogenesis and tyrosine kinase activity in cultured bone marrow fibroblasts by tyrphostins. *Exp. Cell. Res.*, **199**, 255–261.

CHEN, Y.F., PAN, G.Z., HOU, X., LIU, T.H., CHEN, J., YANAIHARA, C. & YANAIHARA, N. (1990). Epidermal growth factor and its receptor in human pancreatic carcinoma. *Pancreas*, **5**, 278–283.

GAZIT, A., YAISH, P., GILON, C. & LEVITZKI, A. (1989). Tyrphostins 1: Synthesis and biological activity of protein tyrosine kinase inhibitors. *J. Med. Chem.*, **32**, 2344–2352.

GAZIT, A., OSHEROV, N., POSNER, I., YAISH, P., PORADOSU, E., GILON, C. & LEVITZKI, A. (1991). Tyrphostins 2: Heterocyclic and α-substituted benzylidenemalononitrile tyrphostins as potent inhibitors of EGF receptor and *erb*B-2/*neu* tyrosine kinases. *J. Med. Chem.*, **34**, 1896–1907.

GILLESPIE, J., POSTON, G.J., SCHACHTER, M. & GUILLOU, P.J. (1992). Human pancreatic cancer cell lines do not express receptors for somatostatin. *Br. J. Cancer*, **66**, 483–487.

KORC, M., MELTZER, P. & TRENT, J. (1986). Enhanced expression of epidermal growth factor correlates with alterations of chromosome 7 in human pancreatic cancer. *Proc. Natl Acad. Sci. USA*, **83**, 5141–5144.

LEMOINE, N.R., HUGHES, C.M., BARTON, C.M., POULSOM, R., JEFFERY, R.E., KLOPPEL, G., HALL, P.A. & GULLICK, W. (1992). The epidermal growth factor receptor in human pancreatic cancer. *J. Pathol.*, **166**, 7–12.

LEVITZKI, A. (1990). Tyrphostins-Potential antiproliferative agents and novel molecular tools. *Biochem. Pharm.*, **40**, 913–918.

LEVITZKI, A. & GILON, C. (1991). Tyrphostins as molecular tools and potential antiproliferative drugs. *T.I.P.S.*, **12**, 171–174.

LYALL, R.M., ZILBERSTEIN, A., GAZIT, A., GILON, C., LEVITZKI, A. & SCHLESSINGER, J. (1989). Tyrphostins inhibit epidermal growth factor (EGF)-receptor tyrosine kinase activity in living cells and EGF-stimulated cell proliferation. *J. Biochem. Chem.*, **264**, 14503–14509.

OHMURA, E., OKADA, M., ONODA, N., KAMIYA, Y., MURAKAMI, H., TSUSHIMA, T. & SHIZUME, K. (1990). Insulin-like growth factor 1 and transforming growth factor α as autocrine growth factors in human pancreatic cancer cell growth. *Can. Res.*, **50**, 103–107.

REDDY, K.B., MANGOLD, G.L., TANDON, A.K., YONEDA, T., MUNDY, G.R., ZILBERSTEIN, A. & OSBORNE, C.K. (1992). Inhibition of breast cancer cell growth *in vitro* by a tyrosine kinase inhibitor. *Cancer Res.*, **52**, 3636–3641.

SMITH, J.J., DERYNCK, R. & KORC, M. (1987). Production of transforming growth factor α in human pancreatic cancer cells: Evidence for a superagonist autocrine cycle. *Proc. Natl Acad. Sci. USA*, **84**, 7567–7570.

YAISH, P., GAZIT, A., GILON & LEVITZKI, A. (1988). Blocking of EGF-dependent cell proliferation by EGF receptor kinases inhibitors. *Science*, **242**, 933–935.

YONEDA, T., LYALL, R.M., ALSINA, M.M., PERSONS, P.E., SPADA, A.P., LEVITZKI, A., ZILBERSTEIN, A. & MUNDY, G.R. (1991). The antiproliferative effects of tyrosine kinase inhibitors tyrphostins on a human squamous cell carcinoma *in vitro* and in nude mice. *Cancer Res.*, **51**, 4430–4435.


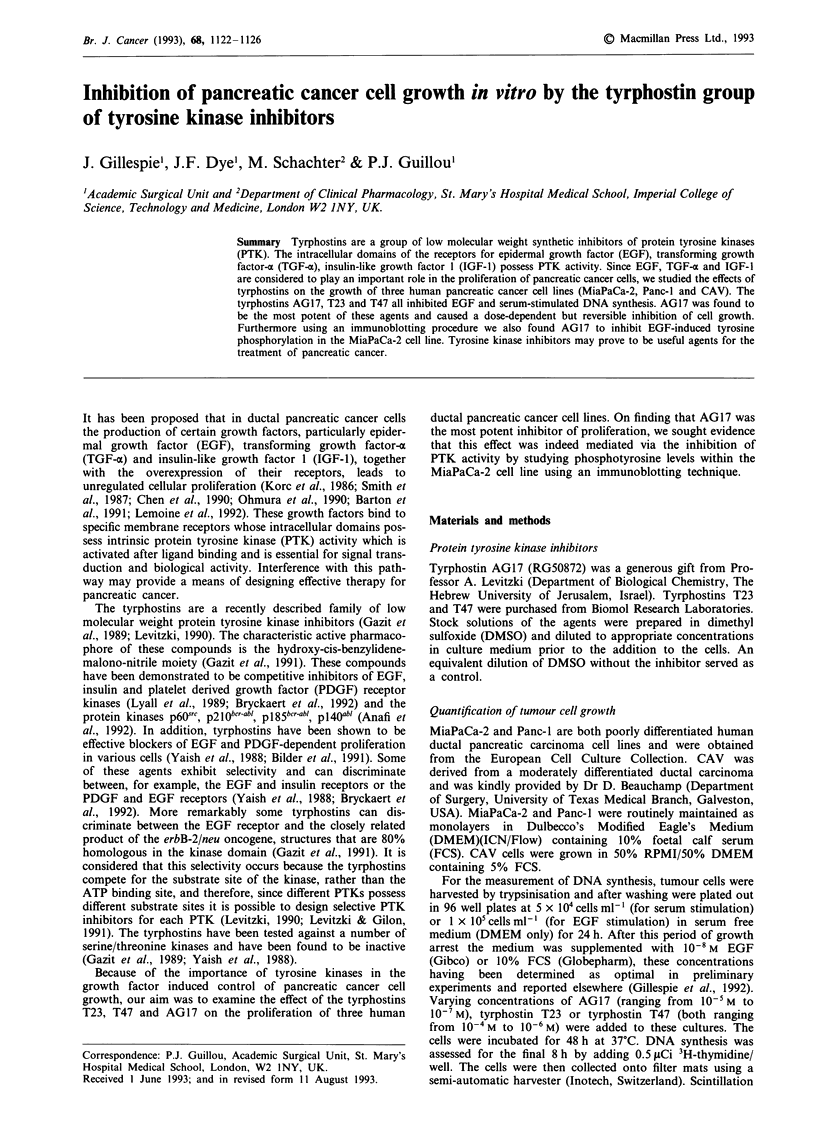

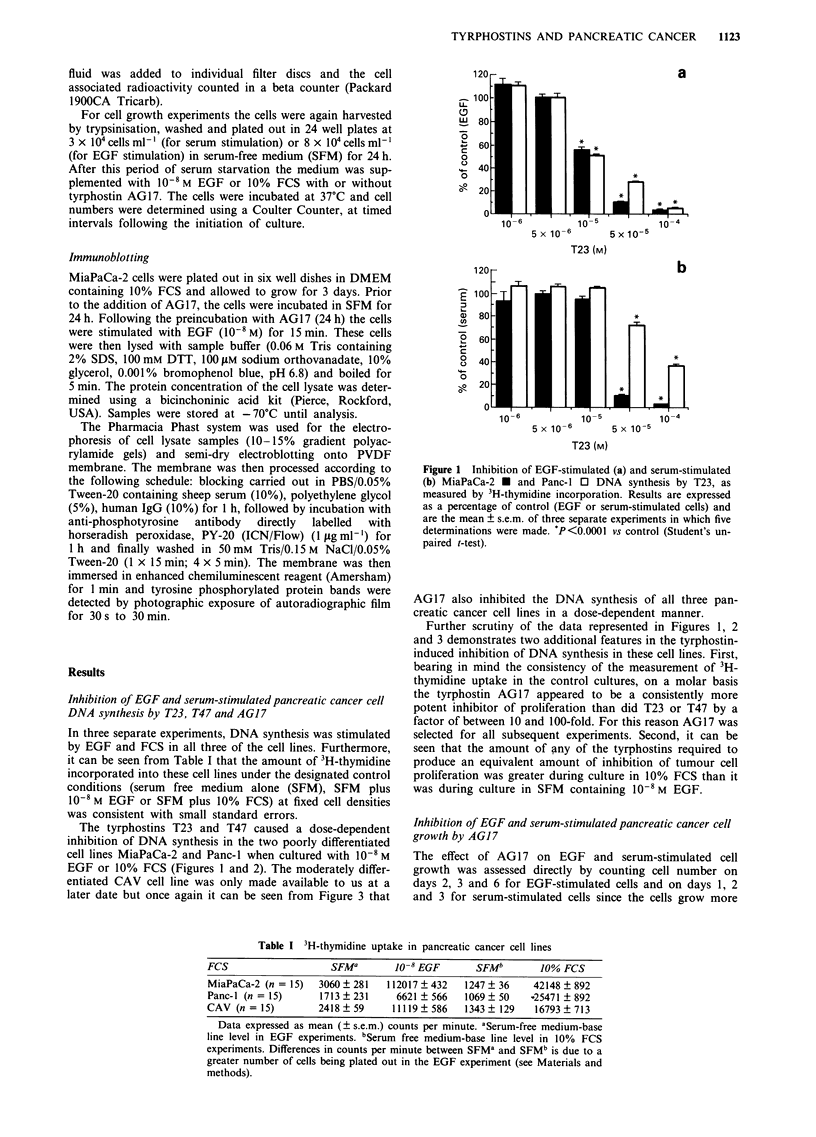

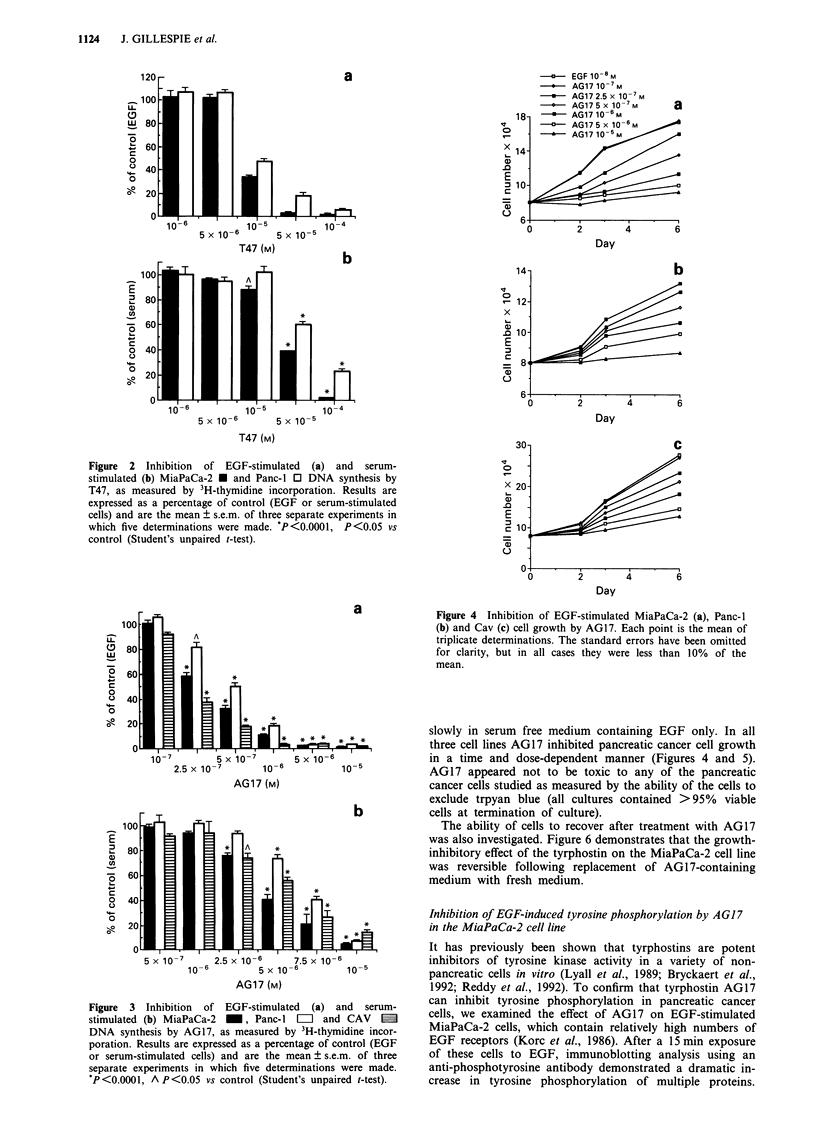

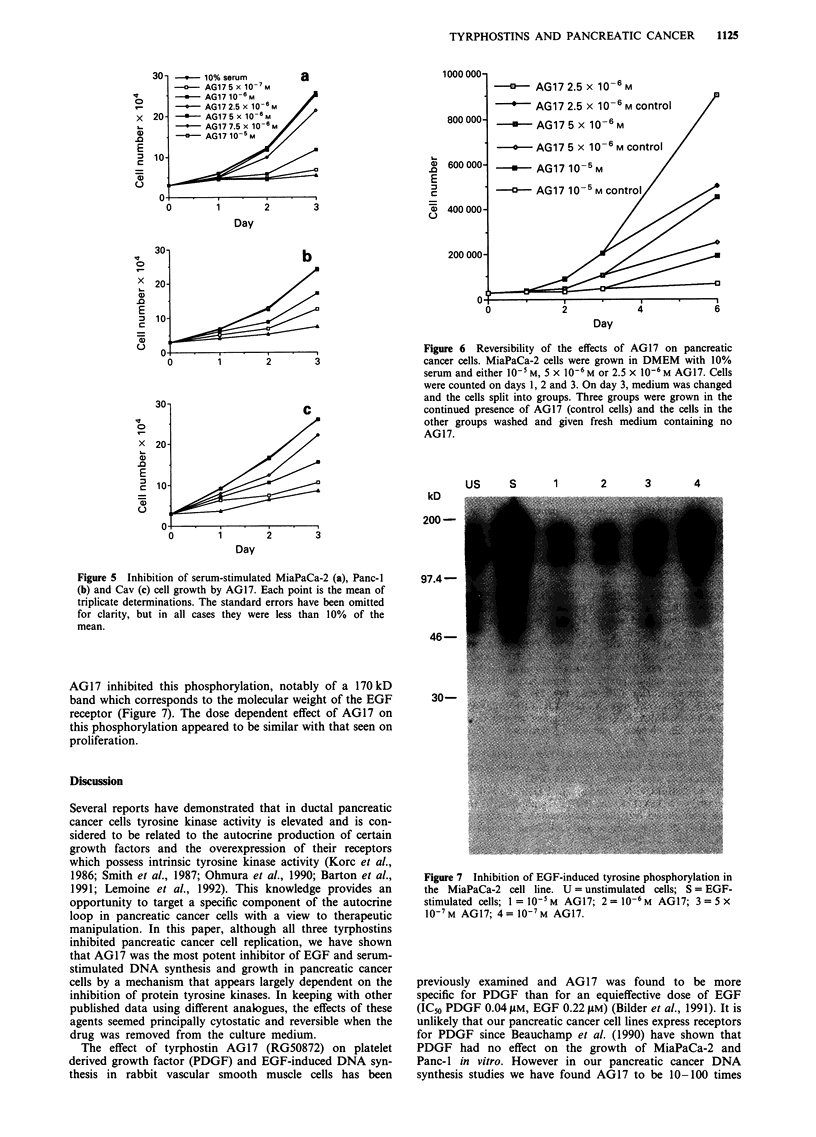

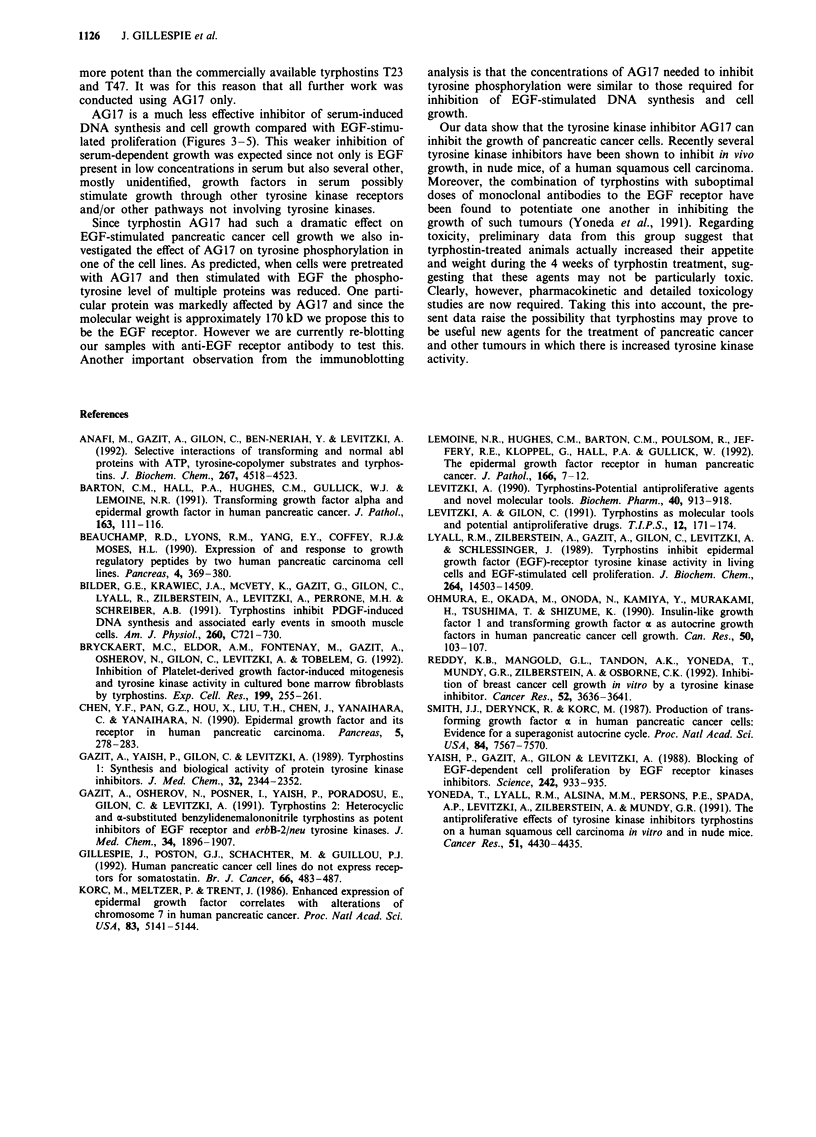


## References

[OCR_00660] Anafi M., Gazit A., Gilon C., Ben-Neriah Y., Levitzki A. (1992). Selective interactions of transforming and normal abl proteins with ATP, tyrosine-copolymer substrates, and tyrphostins.. J Biol Chem.

[OCR_00666] Barton C. M., Hall P. A., Hughes C. M., Gullick W. J., Lemoine N. R. (1991). Transforming growth factor alpha and epidermal growth factor in human pancreatic cancer.. J Pathol.

[OCR_00674] Beauchamp R. D., Lyons R. M., Yang E. Y., Coffey R. J., Moses H. L. (1990). Expression of and response to growth regulatory peptides by two human pancreatic carcinoma cell lines.. Pancreas.

[OCR_00678] Bilder G. E., Krawiec J. A., McVety K., Gazit A., Gilon C., Lyall R., Zilberstein A., Levitzki A., Perrone M. H., Schreiber A. B. (1991). Tyrphostins inhibit PDGF-induced DNA synthesis and associated early events in smooth muscle cells.. Am J Physiol.

[OCR_00685] Bryckaert M. C., Eldor A., Fontenay M., Gazit A., Osherov N., Gilon C., Levitzki A., Tobelem G. (1992). Inhibition of platelet-derived growth factor-induced mitogenesis and tyrosine kinase activity in cultured bone marrow fibroblasts by tyrphostins.. Exp Cell Res.

[OCR_00692] Chen Y. F., Pan G. Z., Hou X., Liu T. H., Chen J., Yanaihara C., Yanaihara N. (1990). Epidermal growth factor and its receptors in human pancreatic carcinoma.. Pancreas.

[OCR_00703] Gazit A., Osherov N., Posner I., Yaish P., Poradosu E., Gilon C., Levitzki A. (1991). Tyrphostins. 2. Heterocyclic and alpha-substituted benzylidenemalononitrile tyrphostins as potent inhibitors of EGF receptor and ErbB2/neu tyrosine kinases.. J Med Chem.

[OCR_00698] Gazit A., Yaish P., Gilon C., Levitzki A. (1989). Tyrphostins I: synthesis and biological activity of protein tyrosine kinase inhibitors.. J Med Chem.

[OCR_00710] Gillespie J., Poston G. J., Schachter M., Guillou P. J. (1992). Human pancreatic cancer cell lines do not express receptors for somatostatin.. Br J Cancer.

[OCR_00715] Korc M., Meltzer P., Trent J. (1986). Enhanced expression of epidermal growth factor receptor correlates with alterations of chromosome 7 in human pancreatic cancer.. Proc Natl Acad Sci U S A.

[OCR_00723] Lemoine N. R., Hughes C. M., Barton C. M., Poulsom R., Jeffery R. E., Klöppel G., Hall P. A., Gullick W. J. (1992). The epidermal growth factor receptor in human pancreatic cancer.. J Pathol.

[OCR_00731] Levitzki A., Gilon C. (1991). Tyrphostins as molecular tools and potential antiproliferative drugs.. Trends Pharmacol Sci.

[OCR_00727] Levitzki A. (1990). Tyrphostins--potential antiproliferative agents and novel molecular tools.. Biochem Pharmacol.

[OCR_00735] Lyall R. M., Zilberstein A., Gazit A., Gilon C., Levitzki A., Schlessinger J. (1989). Tyrphostins inhibit epidermal growth factor (EGF)-receptor tyrosine kinase activity in living cells and EGF-stimulated cell proliferation.. J Biol Chem.

[OCR_00742] Ohmura E., Okada M., Onoda N., Kamiya Y., Murakami H., Tsushima T., Shizume K. (1990). Insulin-like growth factor I and transforming growth factor alpha as autocrine growth factors in human pancreatic cancer cell growth.. Cancer Res.

[OCR_00749] Reddy K. B., Mangold G. L., Tandon A. K., Yoneda T., Mundy G. R., Zilberstein A., Osborne C. K. (1992). Inhibition of breast cancer cell growth in vitro by a tyrosine kinase inhibitor.. Cancer Res.

[OCR_00755] Smith J. J., Derynck R., Korc M. (1987). Production of transforming growth factor alpha in human pancreatic cancer cells: evidence for a superagonist autocrine cycle.. Proc Natl Acad Sci U S A.

[OCR_00761] Yaish P., Gazit A., Gilon C., Levitzki A. (1988). Blocking of EGF-dependent cell proliferation by EGF receptor kinase inhibitors.. Science.

[OCR_00766] Yoneda T., Lyall R. M., Alsina M. M., Persons P. E., Spada A. P., Levitzki A., Zilberstein A., Mundy G. R. (1991). The antiproliferative effects of tyrosine kinase inhibitors tyrphostins on a human squamous cell carcinoma in vitro and in nude mice.. Cancer Res.

