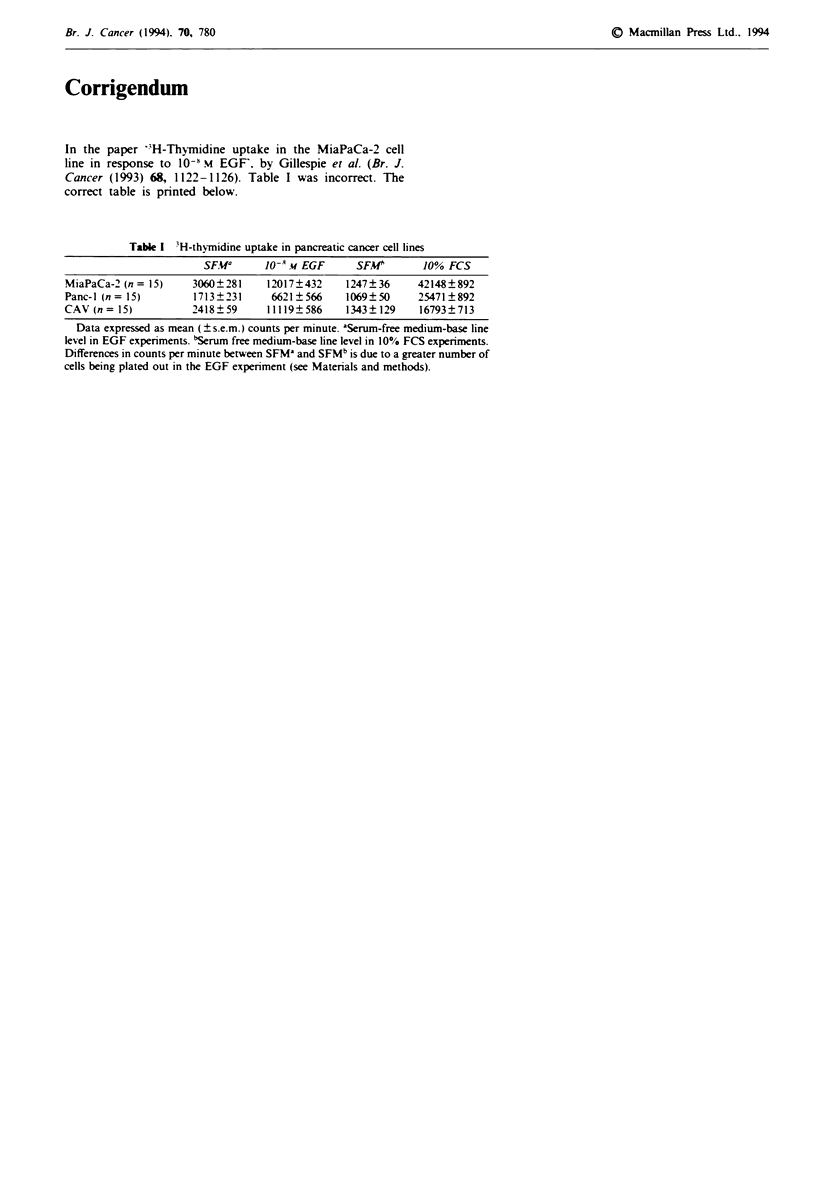# Corrigendum

**Published:** 1994-10

**Authors:** 


 © Macmillan Press Ltd., 1994

### Corrigendum

In the paper '$^3$H-Thymidine uptake in the MiaPaCa-2 cell line in response to $10^{-8}$ M EGF', by Gillespie *et al.* (*Br. J. Cancer* (1993) **68**, 1122-1126). Table I was incorrect. The correct table is printed below.

**Table I** $^3$H-thymidine uptake in pancreatic cancer cell lines

| | *SFM*[a] | *$10^{-8}$ M EGF* | *SFM*[b] | *10% FCS* |
|---|---|---|---|---|
| MiaPaCa-2 (*n* = 15) | 3060±281 | 12017±432 | 1247±36 | 42148±892 |
| Panc-1 (*n* = 15) | 1713±231 | 6621±566 | 1069±50 | 25471±892 |
| CAV (*n* = 15) | 2418±59 | 11119±586 | 1343±129 | 16793±713 |

Data expressed as mean (±s.e.m.) counts per minute. [a]Serum-free medium-base line level in EGF experiments. [b]Serum free medium-base line level in 10% FCS experiments. Differences in counts per minute between SFM[a] and SFM[b] is due to a greater number of cells being plated out in the EGF experiment (see Materials and methods).